# Lipoadenoma of the Parathyroid Gland: Characterization of an Institutional Series Spanning 28 Years

**DOI:** 10.1007/s12022-020-09616-3

**Published:** 2020-03-19

**Authors:** C. Christofer Juhlin, Henrik Falhammar, Jan Zedenius, Inga-Lena Nilsson, Anders Höög

**Affiliations:** 1 Department of Pathology and Cytology, Karolinska University Hospital, Stockholm, Sweden; 2 Department of Oncology-Pathology, Karolinska Institutet, BioClinicum J6:20, 171 64, Solna, Stockholm, Sweden; 3 Department of Endocrinology, Metabolism and Diabetes, Karolinska University Hospital, Stockholm, Sweden; 4 Department of Molecular Medicine and Surgery, Karolinska Institutet, Stockholm, Sweden; 5 Department of Breast, Endocrine Tumors and Sarcoma, Karolinska University Hospital, Stockholm, Sweden

**Keywords:** Parathyroid, Lipoadenoma, Hyperparathyroidism, Case series

## Abstract

Parathyroid lipoadenomas (PLAs) are rare tumors, and case descriptions are limited, < 80 to date. As a consequence, scarce information regarding the etiology of these enigmatic lesions is available. We searched our departmental pathology database for PLAs diagnosed between 1992 and 2020, reexamined the histology of each case, and recorded clinical parameters from the patients’ medical charts. As the diagnostic criteria of this lesion vary over this time period, we defined PLA as an enlarged parathyroid gland with > 50% fat on histologic examination with preoperative evidence of primary hyperparathyroidism (PHPT). A total of 8 bona fide PLA cases and 2 equivocal PLAs (close to 50% fat) were included. As approximately 4000 conventional parathyroid adenomas were diagnosed at our department during the same time interval, the prevalence of PLA was 0.20%. PLA patients were predominately female (63%) and presented with classical PHPT-related symptoms. Majority of cases were successfully located preoperatively and had an average tumor weight of 445 mg. Histologically, all PLAs consisted of > 50% mature adipose tissue, except a single case with brown fat. Of note, PLA patients exhibited a body mass index in line with PHPT patients in general, but a relatively high, near-significant prevalence of arterial hypertension was observed when compared to tumors with less fat (*P* = 0.0584). Future studies on this finding might be warranted. To summarize, we present one of the largest institutional PLA case series to date, and conclude that PLAs are rare, sporadic tumors mirroring many clinical aspects of conventional adenomas—with a potential coupling to hypertension worthy of follow-up studies.

**Electronic supplementary material:**

The online version of this article (10.1007/s12022-020-09616-3) contains supplementary material, which is available to authorized users.

## Introduction

In primary hyperparathyroidism (PHPT), the proper identification of a hyperfunctioning parathyroid gland is often a concerted series of investigations using biochemical, radiological, surgical, and histological approaches. For most instances, a single adenoma is usually identified through laboratory analyses suggesting elevated serum levels of calcium and/or parathyroid hormone (PTH), and preoperative localization techniques may vary among institutions, but most often include neck ultrasound and/or Technetium (99mTc) sestamibi scintigraphy [1, 2]. If the adenoma is pinpointed preoperatively, a focused parathyroidectomy is the preferred strategy as opposed to a more time-consuming bilateral neck exploration of all four glands. Upon histological evaluation, the pathologist relies on the architecture of the excised gland as well as the proportion of stromal fat. A normal gland usually is composed by at least 25% fat, but the proportion might vary with patient age and body habitus [3–5]. Also, the sheer glandular mass is brought into consideration, in which normal glands regularly exhibit weights below 60 mg [6]. Information regarding intraoperative PTH measurements and/or postoperative biochemistry may also constitute useful information, but the picture is complicated by the frequent occurrence of multiglandular disease in PHPT.

Parathyroid lipoadenomas (PLAs) constitute a rare manifestation of PHPT, and few cases have been described in the scientific literature [4, 7–51]. The phenotype was firstly denoted as “parathyroid hamartoma,” but the term “lipoadenoma” gained ground and has since emerged as the most appropriate connotation. The histology bears some similarities to that of a normal parathyroid gland, with a generous proportion of stromal fat and small, labyrinth-like nests of parathyroid cells, mostly chief cells arranged in cords or acini [5]. This makes the distinction between a normal gland and PLA equivocal, and hence some authors propose that the PLA diagnosis should be exclusively used for single adenomas with a clear-cut, postoperative withdrawal of biochemical signs of PHPT [4]. However, this stringent definition could potentially exclude cases in which PLAs develop synchronously with a conventional adenoma, which probably is a not entirely uncommon feature given the proportion of cases of PLAs than do not resolve biochemically after surgical exploration [46].

Other histological features of PLAs include reports of a myxoid, stromal appearance, and, occasionally, the PLA may exhibit admixtures of thymic tissue [8, 9, 11, 17, 28, 33]. A single case has also been reported to display brown fat tissue rather than conventional adipose tissue [51].

From a preoperative perspective, the high adipose tissue content also makes PLAs harder to detect by preoperative imaging techniques compared to conventional adenomas, putting even more emphasis on the clinical enigmas of this tumor subtype [26, 29, 38, 50]. This is not least important, given the fact that subsets of PLAs arise in ectopic locations [41, 42]. Moreover, surgical pathologists who are consulted per-operatively for frozen section diagnosis during parathyroid and thyroid surgery need to be aware of this tumor entity as well. This is especially important when engaged in frozen section assessment of biopsies of parathyroid glands, as the sheer ratio of parathyroid vs stromal fat cells is not entirely reliable to rule out a hyperfunctioning gland [46].

In this study, we sought to characterize the frequency of PLA in a large, institutional series of parathyroid tumors diagnosed in our endocrine pathology tertiary center at Karolinska University Hospital in Stockholm, Sweden. We hereby present one of the largest case series collected and discuss correlations to clinical characteristics of potential interest for future studies.

## Materials and Methods

### Study Cohort and Case Review

We explored the institutional pathology database at Karolinska University Hospital using a general search function using taxonomy based on the Systematized Nomenclature of Medicine (SNOMED) algorithm. The nomenclature chosen was T97*** for parathyroid and M83240 for lipoadenoma. The search was limited from January 1, 1992, to January 31, 2020, as pathology reports prior to 1992 were not incorporated digitally and hence no SNOMED registrations for these cases exist. External consultation cases were excluded to avoid referral bias. All cases retrieved were subsequently retrieved from the pathology repositories and reviewed by conventional light microscopy by two of the authors (CCJ, AH). For this case series, we used a definition in which parathyroid lipoadenoma was denoted as an enlarged parathyroid gland demonstrating a definite increase in parathyroid acinar tissue and > 50% fat on histologic examination excised from a patient exhibiting primary hyperparathyroidism. However, it should be noted that various definitions are used across the scientific literature, and previous reports of this tumor entity reported from a wide variety of institutions only occasionally defined the histological criteria. Moreover, the term “lipohyperplasia” is sometimes used in the literature and usually refers to the synchronous development of several fat-rich enlarged parathyroid glands with our without the postoperative abolishment of hypercalcemia [35, 52]. In our study, patients displaying two or more PLAs synchronously were defined as exhibiting “lipohyperplasia.”

### Clinical Characteristics and Patient Follow-Up

Patients’ medical charts were reviewed thoroughly, and clinical parameters of potential interest were noted for all cases included. Parameters included sex, disease presentation, radiology, complications related to PHPT, tumor weight, co-morbidity, medications, smoking habits, body mass index, age at surgery, surgical complications, length of hospital stay, pre- and postoperative biochemistry, and follow-up time. Outcomes measured were either resolution of PHPT or persistent/recurrent hypercalcemia. Mortality and cause of death was also recorded.

### Institutional Control Cohorts

Data from a clinical control cohort of 110 conventional parathyroid adenomas operated at the Karolinska University Hospital between 2014 and 2018 was used as reference for clinical comparisons with our study. The cohort comprises consecutive cases operated for PHPT at our institution after signed consent. Detailed inclusion and exclusion criteria are available through ClinicalTrials.gov (NCT 02227264). The cohort has been previously published and derives from a registry with comprehensive and biochemical workup, of which we extracted information regarding patient gender, age at surgery, tumor weight, and body mass index (BMI) as well as information regarding persistent or recurrent hyperparathyroidism following surgery [53].

Additionally, to verify that cases annotated as conventional adenomas by SNOMED coding in our institutional series in fact were adenomas with a low adipose tissue proportion and not missed cases of lipoadenomas, we retrieved a total of 200 parathyroid lesions SNOMED coded as adenomas (SNOMED nomenclature M81400) from our tissue archives and reviewed the histology. We refer to this collection as the “histological control group.” These cases were randomly selected from a filtered list of parathyroid adenomas operated at Karolinska between 2016 and 2020, and subsequently matched with the study cohort regarding age at surgery. We then classified the adipose tissue content of each adenoma semi-quantitatively using pre-defined ranges (< 5%, 5%, 10%, 15%, 20%, 25%, or > 25% fat).

### Statistical Analyses

The Mann–Whitney *U*, Kruskal–Wallis, and Fisher’s exact tests were used to compare the tumor groups for differences in clinicopathological variables. *P* values < 0.05 were considered significant. All statistical calculations were performed in SPSS Statistics 26 software (IBM, Armonk, North Castle, New York, USA).

## Results

### Study Cohort

The initial SNOMED search identified 27 tumors from 23 patients, and all pathology files were read manually. After exclusion of erroneous SNOMED coding (*n* = 2) and cases with a suspicion of PLA due to only small areas of the tumor with a generous stromal fat component (*n* = 4, well below the 50% adipose tissue cutoff suggested in our PLA definition described earlier), a total of 21 assumed PLAs from 19 patients were chosen for further scrutinizing. All these cases were retrieved from the tissue archives and reviewed histologically using our abovementioned criteria for PLA. Moreover, all cases were reviewed from a clinical perspective through manual inspection of the patients’ medical records. After exclusion of cases not meeting the histological criteria of PLA (*n* = 10, < 50% fat, *n* = 2, two synchronous PLAs instead meeting our definition of lipohyperplasia) as well as a single case in which slides or blocks were unavailable (*n* = 1), a total of eight cases were finally included as bona fide PLAs. Two cases from the histological exclusion group were re-classified as “equivocal PLA” due to the adipose tissue proportion being close to 50%. The only patient found to exhibit “lipohyperplasia” had two enlarged glands removed with increased fat content, but hypercalcemia persisted. The remaining eight tumors with stromal fat content < 50% were classified as “fat-rich pathological parathyroid glands.”

### Tumor Characteristics

**Fig. 1:**
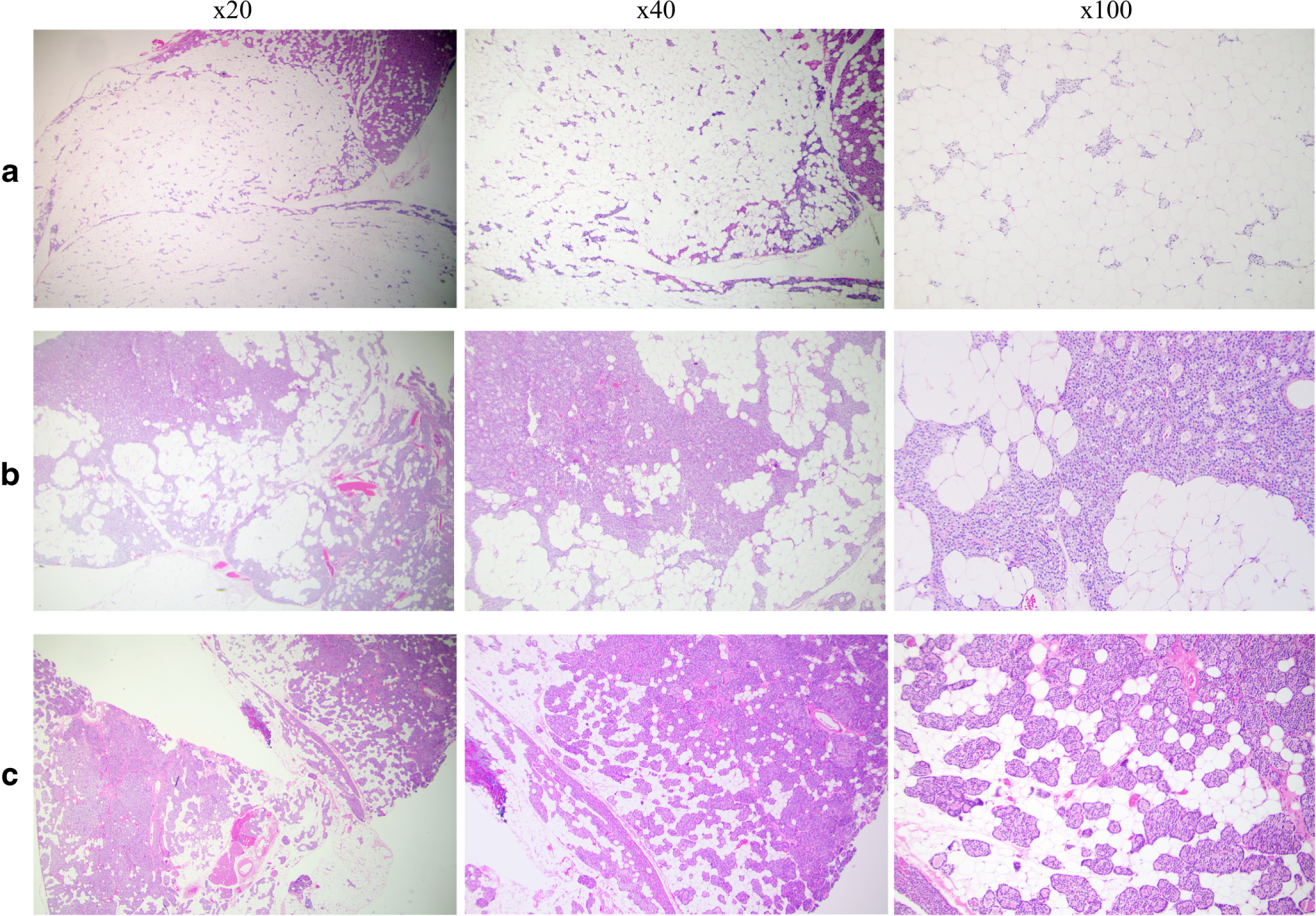
Illustrative photomicrographs of routine hematoxylin-eosin sections magnified × 20 (left column), × 40 (middle column), and × 100 (right column). (A) Parathyroid lipoadenoma (PLA, case 8) demonstrating a rich stromal fat component, > 50% of the tumor proportion. Note the finely dispersed cords of parathyroid cells intermingled in the fat component. A rim of normal parathyroid tissue is evident in the top right corner at lower magnifications. This was the only enlarged gland excised, and the patient’s hypercalcemia resolved postoperatively. (B) Equivocal PLA (case 10) characterized by approximately 50% stromal fat and 50% chief cells. The tumor exhibited a complex heterogeneity, with areas displaying fat cell depletion and areas with abundant adipose tissue, as exemplified in these images. This was the only gland excised, and the patient was normocalcemic postoperatively. (C) Fat-rich pathological parathyroid gland (case 17) with an initial suspicion of PLA given the focal areas enriched for stromal fat. This was the only gland excised, and the patient was cured from primary hyperparathyroidism. Upon histological review, the stromal fat proportion was considered < 50%

The finalized patient cohort consisted of 20 parathyroid glands excised from 18 patients; 8 PLAs from eight patients, two equivocal PLAs from two patients, two glands exhibiting lipohyperplasia in a single patient as well as eight “fat-rich pathological parathyroid glands” cases from seven patients. Summarized clinical and tumor characteristics are detailed in Table 1, and individual information for each case is available as Supplementary Table 1. Examples of histological features of all three groups are illustrated in Fig. 1.

**Table 1:** Summarized clinical and tumor characteristics for the enlisted cases after histopathological and clinical review

Case no.	Age at surgery	Gender	Biochemical evidence of HPT	Glands with unusual fat content	Parathyroid vs adipose tissue proportion (%)	Dx after reevaluation*	Tumor weight (mg)	Additional glands removed^#^ (dx)	Persistent or recurrent HPT^★^	Re-operation (dx)	Resolution of HPT at follow-up
1	47	M	Yes	1	> 50	PLA	900	0	No	No	Yes
2	60	F	Yes	1	> 50	PLA	278	2 (PA, NPG)	No	No	Yes
3	80	F	Yes	1	> 50	PLA	165	0	No	No	Yes
4	82	F	Yes	1	> 50	PLA	490	1 (NPG)	No	No	Yes
5	66	F	Yes	1	> 50	PLA	530	1 (NPG)	No	No	Yes
6	57	M	Yes	1	> 50	PLA	363	0	No	No	Yes
7	61	M	Yes	1	> 50 (brown fat)	PLA	312	1 (NPG)	Persistent	1d (PA)	Yes
8	73	F	Yes	1	> 50	PLA	525	0	No	No	Yes
9	53	F	Yes	1	Approx. 50	Equivocal PLA	286	0	No	No	Yes
10	74	F	Yes	1	Approx. 50	Equivocal PLA	322	0	No	No	Yes
11	50	M	Yes	2	> 50	Lipohyperplasia	115/180	1 (NPG)	Persistent	No	No
12	52	F	Yes	1	> 25–< 50	FRPPG	325	0	No	No	Yes
13	80	M	Yes	1	> 25–< 50	FRPPG	281	0	No	No	Yes
14	61	F	Yes	1	> 25–< 50	FRPPG	183	1 (PA)	No	No	Yes
15	61	F	Yes	1	> 25–< 50	FRPPG	209	0	Persistent	18m (PA, NPG)	Yes
16	39	F	Yes	1	> 25–< 50	FRPPG	222	0	No	No	Yes
17	67	F	Yes	1	> 25–< 50	FRPPG	363	0	Recurrent	No	No
18	72/73	M	Yes	2	> 25–< 50	FRPPG	155/119	1 (PA) ○	Persistent	No	No

*HPT* hyperparathyroidism, *dx* diagnosis, *mg* milligrams, *M* male, *F* female, *PLA* parathyroid lipoadenoma, *FRPPG* fat-rich pathological parathyroid gland, *PA* conventional parathyroid adenoma, *NPG* normal parathyroid gland, *approx*. approximately, *d* day/s, *m* month/s

*Histopathological re-examination using defined criteria of > 50% adipose tissue in an enlarged gland as diagnostic for PLA

^#^Refers to additional parathyroid glands surgically excised synchronously with the pathological gland exhibiting an unusual fat content

^★^Persistent and recurrent HPT defined as elevated calcium and/or PTH detected < 6 and > 6 months after surgery for HPT respectively

^○^Refers to the second round of surgery, in which a FRPPG was removed together with a PA at the age of 73

**Fig. 2:**
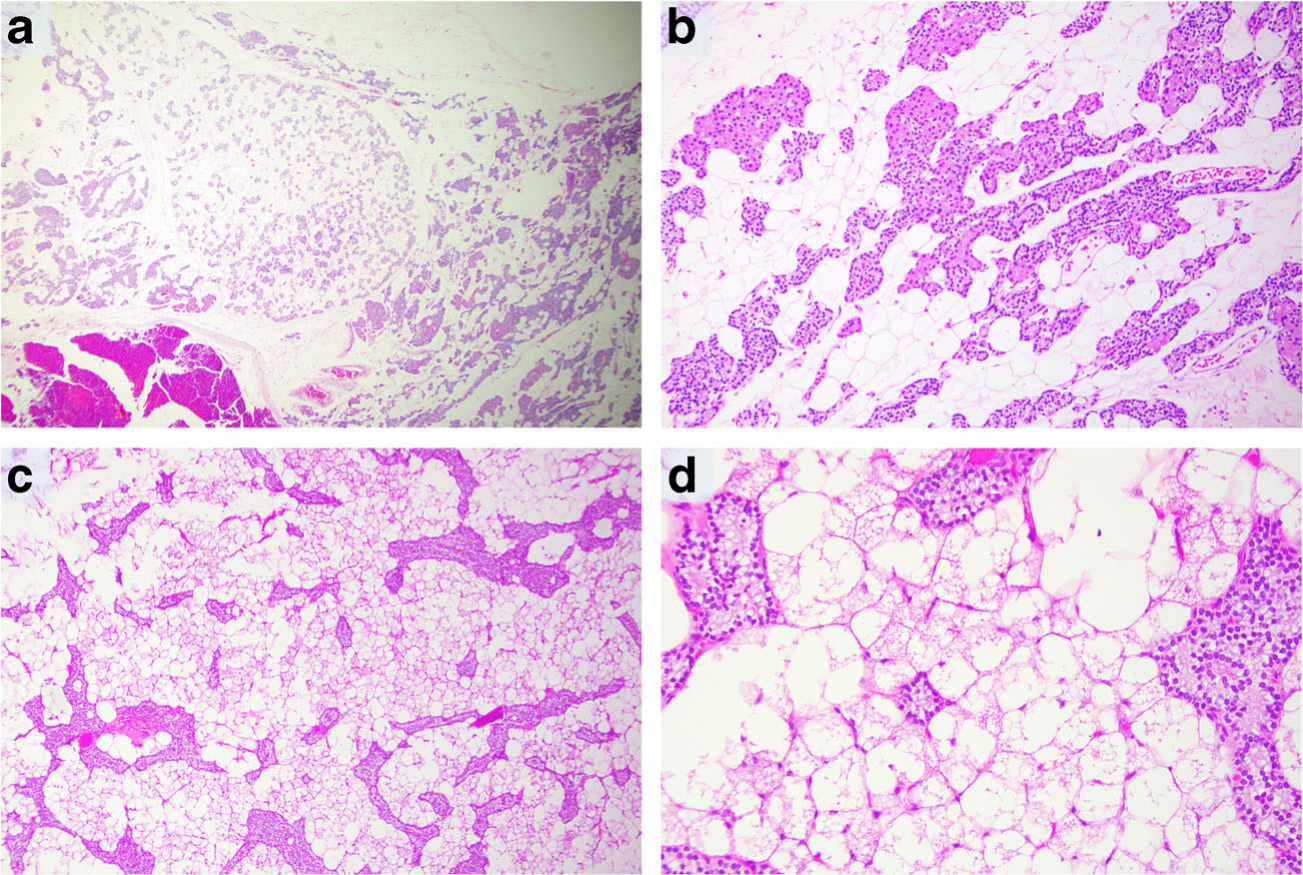
Two separate types of adipose tissue in parathyroid lipoadenomas. **a**, **b** Conventional parathyroid lipoadenoma (PLA, case 2) built up by > 50% mature adipose tissue, with branching cords of chief cells and occasional oxyphilic cells embedded in the stroma. Magnification level × 20 and × 100 respectively. This phenotype was seen in 7 out of 8 PLAs. **c**, **d** PLA with brown fat (case 7), believed to be the second case reported in the scientific literature. Note the eosinophilic appearance of the mitochondria-rich brown adipocytes with parathyroid chief cells intermingled. Magnification level × 40 and × 200 respectively

No case with an ectopic PLA location was noted. The weight of the PLAs ranged from 165 to 900 mg, with a mean weight of 445 mg (Table 1). All PLAs exhibited > 50% proportion of stromal fat with chief cells and occasional oxyphilic cells arranged in branching cords (Fig. 1). No myxoid appearance was noted. One single case (case 7) displayed an abundance of brown fat rather than conventional mature adipose tissue (Table 1, Fig. 2). In all cases, the parenchymal component was free of nuclear pleomorphism and nucleolar prominence. Two cases (cases 1 and 3) were diagnosed by intraoperative frozen sections and subsequently confirmed by assessment of FFPE material, while the remaining cases were diagnosed postoperatively using FFPE material exclusively (Supplementary Table 1). All cases except one (case 7) displayed resolution of primary hyperparathyroidism following the initial surgery. Case 7 was re-operated 1 day later due to persistent hypercalcemia, and a conventional parathyroid adenoma was excised—which cured the patient.

**Fig. 3:**
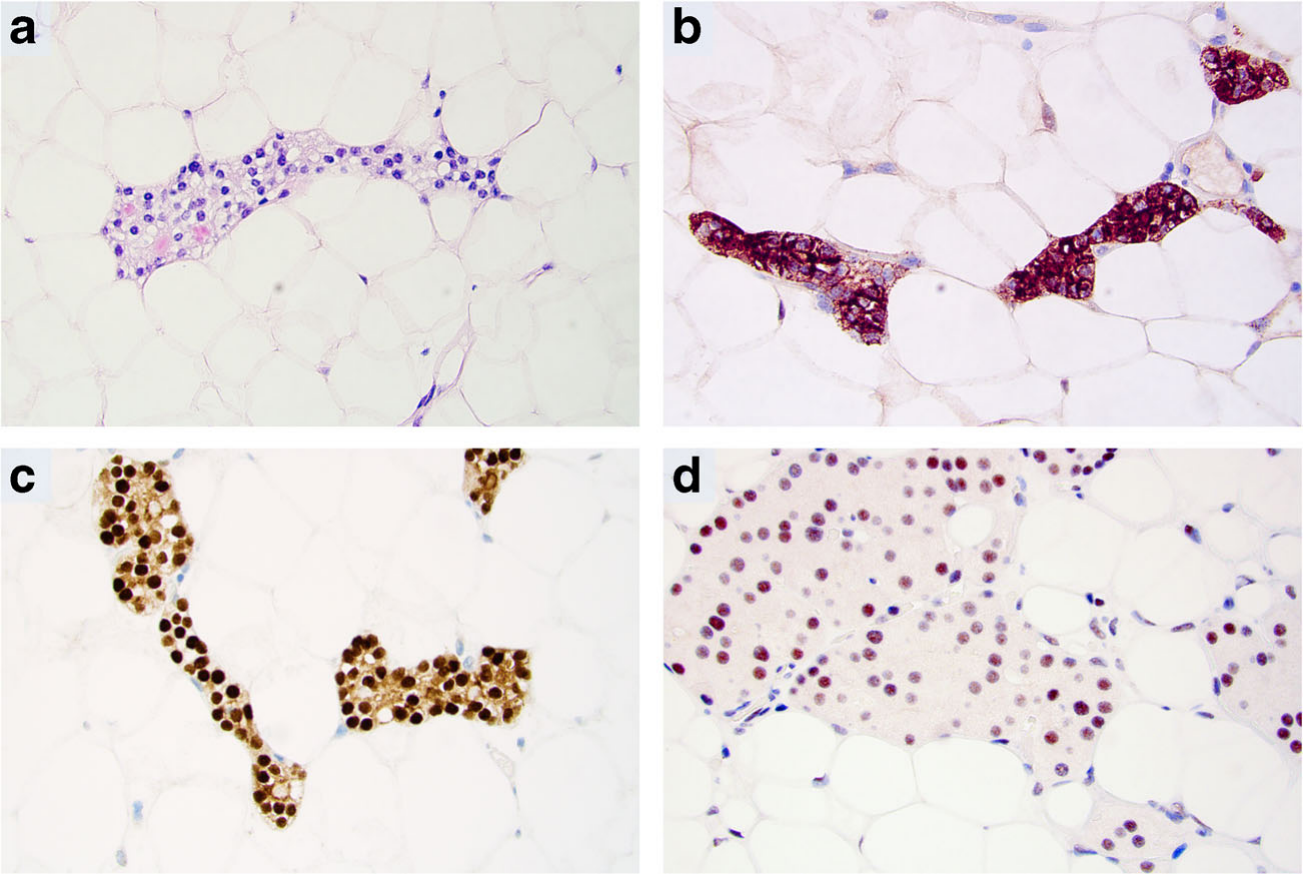
Immunohistochemical profile of parathyroid lipoadenomas (PLAs). All photomicrographs are magnified × 400. **a** Routine hematoxylin-eosin stain of PLA (case 8), showcasing a group of chief cells embedded in mature adipose tissue. Subsequent images display **b** cytoplasmic PTH immunoreactivity, and **c** GATA3 nuclear immunoreactivity as well as **d** retained nuclear parafibromin immunoreactivity

Immunohistochemistry was ordered for a single PLA case (case 8, Fig. 3). No cases were diagnosed as atypical adenomas or parathyroid carcinomas.

Two cases (cases 9 and 10) were named “equivocal PLAs” due to the adipose tissue content reaching approximately 50%, making it hard to exclude them from a PLA diagnosis for this reason—as the 50% cutoff is arbitrarily set (Table 1, Fig. 1). Both patients were normocalcemic on follow-up, thereby supporting our suspicion that these lesions were in fact functional.

Two removed glands from a single patient (case 11) displayed > 50% fat content, and the patient displayed persistent hypercalcemia postoperatively (Table 1). The patient was excluded from the PLA category as he exhibited two enlarged glands, and hence the diagnosis of “lipohyperplasia” was recommended.

The remaining eight glands from seven patients not fulfilling the histological criteria for PLA are detailed herein as a comparative group entitled “fat-rich pathological parathyroid glands,” as they exhibit stromal fat proportions above the usual 25% seen in normal glands (Table 1, Table 2, Fig. 1). The mean tumor weight of this group was 232 mg (range 119–363 mg), significantly smaller than the PLA group tumors (Mann–Whitney *U*, *P* = 0.0356). Of these patients, four were cured biochemically after first surgery. One additional patient was cured after a re-operation 1.5 years later, in which an intrathyroidal parathyroid adenoma was discovered (case 15). Two additional patients are still not cured biochemically, but asymptomatic and hence only followed (cases 17 and 18).

**Table 2:** Semi-quantitative assessment of fat content in parathyroid tumors from the study and histological control cohorts

Adipose tissue content*								
	< 5% fat	5% fat	10% fat	15% fat	20% fat	> 25–< 50% fat	50% fat	> 50% fat
Study cohort cases								
PLAs (*n* = 8)	0	0	0	0	0	0	0	8
Equivocal PLAs (*n* = 2)	0	0	0	0	0	0	2	0
Lipohyperplasia (*n* = 2)	0	0	0	0	0	0	0	2
FRPPGs (*n* = 8)	0	0	0	0	0	8	0	0
Histological control cohort								
PAs (*n* = 200)	149	20	18	11	2	0	0	0

*PLAs* parathyroid lipoadenomas, *FRPPGs* fat-rich pathological parathyroid glands, *PAs* conventional parathyroid adenomas

*Mature adipose tissue, with the exception of case 7 (PLA) in which brown fat cells were estimated

### Adipose Tissue Proportions Compared to the Histological Control Group

As a reference group, we reviewed the histology of 200 age-matched conventional parathyroid adenomas diagnosed at our institution in terms of adipose tissue content semi-quantitatively using pre-defined ranges (< 5%, 5%, 10%, 15%, 20%, 25%, or > 25% fat), and the results are detailed in Table 2. Most conventional adenomas exhibited < 5% adipose tissue (*n* = 149; 74.5%), and subsets of cases were found to exhibit around 5% (*n* = 20; 10%) and 10% (*n* = 18; 9%) of fat respectively. Eleven cases demonstrated around 15% fat content (5.5%), and only single cases displayed an adipose tissue content of 20% (*n* = 2; 1%). No case exhibited an adipose tissue proportion above 20%, which should be compared to the 25% cutoff used for fat-rich pathological parathyroid glands in our study.

### Coupling to Clinical Parameters

The eight PLA patients were predominantly female (63%), and median age at surgery was 63.5 years (range 47–82) (Table 1). The follow-up time ranged from 2 to 14 years (median 10.5 years). No patient exhibited family history indicative of PHPT. All patients were non-smokers except one. Most PLA patients had mild PHPT with classical symptomatology. Four out of four informative cases were osteopenic or osteoporotic. The tumor was for most cases identified using both ultrasound and scintigraphy. Surgery was largely uneventful, and patients were generally discharged day 1 postoperatively (Table 1, Supplementary Table 1).

Given the relationship between body habitus and stromal fat content of parenchymal tissues, we wanted to examine the BMI of all study patients. For the bona fide PLAs, the median BMI was 25.9 kg/m^2^ (range 20.7–49.5), and for the “fat-rich pathological parathyroid glands” group, the median BMI was 26.8 kg/m^2^ (range 20.2–30) (Supplementary Table 1). As a comparison, the clinical control cohort of 110 conventional parathyroid adenomas displayed a mean BMI of 26.6, with a median of 25.9. We found no evidence of a statistically significant difference between the clinical control cohort and the merged PLA/equivocal PLA group in terms of body composition (Mann–Whitney *P* = 0.403, Table 3). In fact, no significant difference in BMI was noted when performing a multi-group comparison either (clinical control cohort vs PLAs vs equivocal PLAs vs lipohyperplasia vs fat-rich pathological parathyroid glands (Kruskal–Wallis *P* = 0.781)).

**Table 3:** Clinical comparisons between the study and clinical control cohorts

	Gender (M/F)	Age at surgery (mean/median)	Tumor weight in mg (mean/median)	BMI (mean/median)	Recurrent/persistent HPT (events/total)
Study cohort patients					
PLAs and equivocal PLAs (*n* = 10)	3/7	65.3/63.5	417.1/342.5	29.3/26.1	5/18
Lipohyperplasia (*n* = 1)^**#**^	1/0	–	–	–	
FRPPGs (*n* = 7)	2/5	61.7/61.0	248.3/222	26.0/26.8	
Clinical control cohort					
PAs (*n* = 110)	19/91	63.1/62	654.6/355	26.6/25.9	3/110
*P* value *	*P* = 0.389	*P* = 0.575	*P* = 0.904	*P* = 0.403	*P = 0.001*

*PLAs* parathyroid lipoadenomas, *FRPPGs* fat-rich pathological parathyroid glands, *PAs* conventional parathyroid adenomas

^**#**^Single case, hence excluded from all analyses except for recurrence/persistent HPT

*P values were obtained from comparisons between PLAs/equivocal PLAs vs the clinical control cohort, except for the comparison of recurrences, which include all groups

All statistically significant *P* values are marked in italics

We also analyzed additional parameters between the study cohort and the clinical control cohort, and found that the merged PLA/equivocal PLA group was similar to the conventional adenomas from the clinical control cohort in terms of gender distribution (Fisher’s exact test, *P* = 0.389), age at surgery (Mann–Whitney *P* = 0.575), and tumor weight (Mann–Whitney *P* = 0.904) (Table 3).

Six out of eight PLA patients (75%) and both patients with equivocal PLA (100%) presented with arterial hypertension compared to two out of seven the “fat-rich pathological parathyroid glands” group (29%) (Supplementary Table 1). This difference between the two former and the latter group did not reach a statistical significance (Fisher’s exact test, *P =* 0.0584), but a trend was noted even though the patient numbers are scarce.

We also analyzed the clinical control cohort for the prevalence of persistent or recurrent hyperparathyroidism and compared the findings to our own cohort of parathyroid glands with increased fat. We found that our study cohort in general displayed a higher proportion of patients with relapsing/persistent hyperparathyroidism compared to the clinical control cohort (5 events in 18 cases in our study cohort vs. 3 out of 110 in the reference population (Fisher’s exact test, *P* = 0.001, Table 1 and Table 3)).

Regarding the occurrence of unrelated tumors in our study cohort, two PLA patients were diagnosed with breast cancer and follicular thyroid adenoma, respectively. One patient exhibiting an equivocal PLA exhibited a medical history including a synovial chondroma, and one patient from the “fat-rich pathological parathyroid glands” group was diagnosed with breast cancer (Supplementary Table 1). No mortality was recorded among the PLA patients, but one patient with equivocal PLA died 1 year after surgery due to a hematological disorder. One of the patients from the “fat-rich pathological parathyroid glands” cohort deceased after 6 years from a cardiovascular event.

### Literature Search

After reviewing PubMed/MEDLINE repositories and conducting separate searches using standard web browsers for journals not indexed in the aforementioned databases, we found available and readable descriptions of 72 cases of PLA up until February 2020 [4, 7–51]. Comprehensive summaries have been reported elsewhere, the latest in 2016 [46]. The definition of PLA has changed over time, including the amount of mature adipose tissue needed for the diagnosis. Moreover, methods for measuring serum calcium and/or PTH have varied over the years, and not all cases have reverted biochemically after parathyroid surgery, highlighting the complexity of searching the literature for relevant cases.

From the current literature, it seems evident that the majority of PLA cases are female patients exhibiting classical features of PHPT, in which the excision often leads to the reversion of hypercalcemia and/or elevated PTH levels. No case with malignant features (perineural or vascular invasion, invasion of adjacent tissues, distant metastases) has been described. The underlying genetics are almost entirely obscure, and an eventual coupling to other intrinsic or extrinsic factors has not been established.

## Discussion

Although the main bulk of parathyroid tumors is routinely diagnosed by conventional histology, specific subgroups demand increased attention. This is particularly true for parathyroid carcinoma and the borderline group “atypical adenoma,” of which auxiliary immunohistochemical and genetic analyses have facilitated the detection of cases exhibiting malignant potential [54, 55]. Another troubling aspect of diagnosing parathyroid tumors revolves around the proper identification of PLAs and separating them from normal parathyroid glands, not least in an intraoperative diagnostic setting using frozen sections. As the amount of reported PLAs across the scientific literature is exceedingly low (< 100 cases), detailed descriptions of large case series with coupling to clinical parameters are warranted.

In our material, we confirm earlier observations that PLA patients exhibit classical features of PHPT, with modest symptoms related to hypercalcemia [4, 46]. Patients were largely postmenopausal females exhibiting single adenomas with the postoperative resolution of hypercalcemia, with similar age, tumor weight, and BMI as patients with conventional adenomas. No PLA case in our series displayed evidence of atypia or features associated to parathyroid carcinoma. We furthermore acknowledge the rarity of these lesions, with a prevalence of 0.20% in our material. Interestingly, no PLA case was detected in our databases prior to 2005, although the search was conducted in material from 1992 and onwards. The reason for this observation could be previous inadequate SNOMED coding of rare variants, and/or a simple question of sample volumes, as parathyroid surgery volumes rose significantly around 2005 following a departmental concentration of all endocrine surgery in Stockholm.

Histologically, a single case (case 7) was built up by an abundance of brown adipocytes with an associated cord-like distribution of parathyroid chief cells. This patient was a 61-year-old male displaying clinical signs of depression as well as elevated serum calcium and plasma PTH levels. To our knowledge, this is the second description of such a brown fat PLA available in the literature [51].

No clear-cut association between the development of PLAs and patient BMI was noted, although the restricted number of patients in this case series is a clear limitation to these observations. Nevertheless, the PLA group exhibited a median BMI of 25.9 kg/m^2^ (range 20.7–49.5). Consulting previous BMI results of unselected Swedish PHPT patients (*n* = 150), a median BMI value of 26 kg/m^2^ has been reported, which is in line with data from our PLA cohort [56]. Further comparisons with a clinical control cohort of 110 cases yielded no significant correlation either, and hence an eventual association to body composition would have to be sought in a larger material, preferably through a comprehensive meta-analysis.

Regarding arterial hypertension, the vast majority of PLA and equivocal PLA patients were hypertensive and received anti-hypertensive drugs. In the scientific literature, PHPT is tightly coupled to hypertension, and 35–55% of PHPT patients have been reported to suffer from this condition [57]. Although the results from our limited PLA cohort might be a reflection of sampling bias, the potential association could be of clinical importance worthy of attention in subsequent studies of hypertension and hypertensive medications in the PHPT population.

Two PLA patients (two females, 53- and 75-year-old) in our series exhibited borderline tumors in terms of histological presentation and were therefore separated for most analyses. Intriguingly, one of these patients exhibited a positive family history, with two first-degree relatives with PHPT. Unfortunately, these family members were lost to follow-up, and no investigations could be performed as to whether their tumors also were lipomatous. A single case of “lipohyperplasia” was also noted. This male patient was 50 years old at surgery and was excluded from the PLA group based on the findings of two enlarged glands. He is still hypercalcemic and asymptomatic, but followed biochemically. He exhibited a BMI of 25.1 kg/m^2^ at surgery and had no family history indicative of PHPT or additional medical history, except for successful treatment of a deep vein thrombosis.

In this study, we chose to include the eight parathyroid lesions from seven patients that were originally suspected to constitute lipoadenomas, but failed inclusion after histological review. We appointed these cases as “fat-rich pathological parathyroid glands” and found that they were significantly smaller than the PLAs of this cohort. These parathyroid glands were all enlarged but exhibited a stromal fat content of < 50%, but above 25%. Even though the stromal fat proportion was higher than usually seen in conventional adenomas, the enlarged gland made the pathologist reluctant of diagnosing these glands as normal—which was probably correct given the resolution of hypercalcemia in the majority of patients. To support this, conventional adenomas at our institution were consistently fat-depleted when reviewed histologically, and not a single case of the 200 tumors re-evaluated displayed an adipose tissue proportion of > 25%. Interestingly, recent findings from Australia suggest the occurrence of a histological subgroup which the authors entitled “large normal” parathyroid glands (LNPs)—defined by an enlarged parathyroid gland with near-normal histology followed by the postoperative resolution of hypercalcemia [58]. The prevalence of LNP in that specific study was 2%, compared to the PLA prevalence of < 0.5% in our series—making the former an entity of certain clinical impact, as several cases are expected annually in parathyroid high-volume centers. It seems as though our group of so-called fat-rich pathological parathyroid glands bear many similarities with the definition of LNPs, and might indicate a subgroup within the PHPT family of disease entities.

Interestingly, three out of seven patients with a fat-rich pathological parathyroid gland in our study did not resolve biochemically; one case resolved 18 months postoperatively after the excision of a conventional adenoma, and the remaining two are still not cured. Indeed, the study cohort displayed significantly worse clinical outcome in terms of resolution of hyperparathyroidism compared to the clinical control cohort. This highlights the intricacy of these patients from a clinical perspective and might signify that several patients with fat-rich parathyroid lesions exhibit clinically elusive multiglandular disease.

In all, it seems safe to conclude that a proportion of pathological parathyroid lesions in patients with PHPT is hard to detect histologically by means of tumoral weight and stromal fat content only. Hence, more emphasis on a holistic process of diagnosing these lesions seems appropriate, in which histology and postoperative, biochemical workup is merged. Future studies might help dissect whether there is a continuous spectrum of disease development, from a normal gland, via LNPs to PLAs and/or conventional adenomas, or if the different entities are branched with associated, underlying exogenous factors and/or genetic aberrancies governing the development of each unique group. Indeed, future meta-analyses of PLAs might bring new light to the possible etiologies of these lesions, including the observed association with hypertension described here.

## Electronic Supplementary Material

ESM 1 (XLSX 14 kb)
